# 

**Published:** 1892-03

**Authors:** 


### CONTENTS.

ORIGINAL COMMUNICATIONS—

Twenty-eight Consecutive Laparotomies. By Virgil O. Hardon, M. D., Atlanta, Ga. .... 1

Cystitis. By C. E. Murphy, M. D., Atlanta, Ga. .... 11

"Laparotomies Performed During the Past Year." (Opie.) By W. E. B. Davis, M. D., Birmingham, Ala. .... 18

"Ringworm." By M. B. Hutchins, M. D., Atlanta, Ga. .... 20

SOCIETY REPORTS—

The Atlanta Society of Medicine .... 26

Discussion on Dr. Brown's Paper. (S. S. and G. Association.) .... 36

CORRESPONDENCE—

Items of Medical Interest in New York .... 44

EDITORIAL—

"The Bearing of Recent Biological Researches on the Practice of Medicine and Surgery." .... 50

Europhen .... 55

Closing of the Medical Schools .... 56

SELECTIONS .... 58-60

MEDICAL ITEMS .... 61-62

BOOK REVIEWS .... 63-64

INDEX TO ADVERTISEMENTS .... xiii

ITEMS OF INTEREST .... xi and xii

CLUB RATE PAGE .... xiv

